# Supplementary material for: The effect of script reform on levels of orthographic knowledge: Evidence from alphasyllabary Malayalam scripts
Source: PLoS One. 2023 Aug 3;18(8):e0285781. doi: 10.1371/journal.pone.0285781 (PMC10399769; doi:10.1371/journal.pone.0285781)
Supplement: Attachment — Submitted filename: Review.pdf [file pone.0285781.s001.pdf]

The paper presents a very interesting processing study that has a cultural as well as historical aspect to it. I recommend it for publication. The study looks at whether orthographic knowledge is affected by changes (institutionalised reform) in the standard orthography of a language. Specifically, the paper looks at how the Malayalam script reform in 1971 affected the lexical and sublexical orthographic knowledge in the users of the script. The chronology of the reform allowed the authors to have a pre-reform and a post-reform group. The expectation was that the pre-reform group would take longer to read the reformed script (RS) and the post reform, the traditional script (TS). The participants were also given a production task, specifically a writing task, where they were asked to write down sentences that were dictated to them. I must mention at this point that the script reform was intended as a solution to 'redundant complex grapheme' and an absence of space in the typical manual typewriter of the time. It was predicted that the pre-reform group would choose to write in TS and the post-reform group in RS. Findings showed that both groups were faster in reading RS; and for the writing task, the pre-reform group continued to use TS but the post-reform group wrote in RS.

The results are very interesting, and the analysis provided was enlightening. The reason for both groups being faster was attributed to longer exposure to RS script post 1971. This is taken to imply that lexical orthographic knowledge is flexible depending on exposure. Then the question is, why is this pattern not observed in the writing task? This is where sublexical orthographic knowledge comes into play; the study suggests that sublexical rules ‘seem’ rigid, although their rigidity may be due to a lack of writing practice in the age of digital media. Even if you are a fluent reader of RS, if you learnt TS in school, you might choose TS. This is because writing is also a production task, which may be affected by writing practice. The correlation between group type and script type is taken as further evidence for this type of analysis. An important conclusion that the paper arrives at is the confirmation that orthographic knowledge consists of two independent but related levels: lexical and sublexical orthographic knowledge.

Additional interesting points from the paper:
1. The paper provides a good overview of certain characteristics of Malayalam orthography that can have consequences in terms of language processing, such as complex glyphs. Additionally, it also provides the historical context and contemporary relevance for orthography reforms.
2. The paper also explains how experimental methodologies such as self-paced reading tasks and writing tasks differ; the former is a perception-based task whereas the latter is a production task. The suggestion that a lack of exposure in the form of practice affects production tasks, as they generally incur more processing load is significant.

Points to improve/Suggestions for further research:

1. In section 6 (lines 414-415), the paper states that the correlation between group type and script type confirms that sublexical knowledge is determined by the script one learns in elementary education. However, does this correlation imply causation or confirmation? This question is significant because the later discussion mentions that sublexical orthographic knowledge could be restructured through continued exposure in the form of writing practice. There seems to be some conflict in clarity there.
2. In addition to writing being a production skill, it also differs from reading in terms of modality. Writing also has a motor processing component to it in addition to retrieval of orthographic representations. The paper briefly mentions age being a possible factor in writing speed in older participants. Is it possible for this difference in modality to have other consequences when it comes to the choice or preference of script type apart from exposure/writing practice?
3. It would have been helpful if lexical and sublexical orthographic knowledge were explained using examples/language data.
4. Similarly, more explanation for certain memory and processing related terms such as *retrieval* would be helpful for a reader who may be new to the area.
5. Is it possible that changes made in the script reform were also keeping in view that these revisions would simplify the script in terms of the cognitive load on the user? The paper passingly suggests otherwise. However, do you think it would be right to assume that reforms with such pragmatic intentions would naturally result in a simpler yet more efficient orthographic system?

6. I would also like to alert the authors of some minor proof-reading errors that need to be corrected.
