# Supplementary material for: The effect of script reform on levels of orthographic knowledge: Evidence from alphasyllabary Malayalam scripts
Source: PLoS One. 2023 Aug 3;18(8):e0285781. doi: 10.1371/journal.pone.0285781 (PMC10399769; doi:10.1371/journal.pone.0285781)
Supplement: Attachment — Submitted filename: Response to reviewers.docx [file pone.0285781.s002.docx]

We would like to thank the reviewers for their thought-provoking and helpful remarks. The reviewers have pointed out very valid concerns that needed our attention and we were able to work on those areas. The reviewers’ remarks are in black font colour. Our responses are underneath each remark in red font colour. The changes have been made using *track change* in the manuscript in various places. 

**Replies to Reviewer 1 comments:**

The following are some of the concerns that needs to be reviewed:

In the data provided (uploaded Excel sheet), the classification of word type – Complex/ Non-Complex is confusing. In the study, you have mentioned that;
“The sentences had total 59 words with complex glyphs, such that each sentence had at least four instances of the complex glyphs.”
Please elaborate the premise you used to characterize certain glyphs as complex glyphs.

Additionally, the classification in terms of Word Type requires further justification as in some sentences, 1 or 2 words are classified as ‘Complex’ Word Type while in other sentences, there are NO words that are classified as ‘Complex’ Word Type.

Glyphs from TS with conjoined conjuncts and vowel notations attached to the consonants and conjuncts (Fig 1) are classified in the study as complex glyphs. The 50 Malayalam sentences had at least four instances of these complex glyphs out of which words with two or more than two complex glyphs in them were classified as the Complex Word Type. A sentence from the experiment set, thus, would have complex glyphs but not always the Complex Word Type. We have elaborated and rewritten this section in the manuscript (lines 257-269).


Additional concerns with LINE number:

Line 350-353
“We checked the correlation between the age group of the participants and the RS glyphs written by them using the Pearson's product-moment correlation. The r value was 0.73. The r value between the age group of participants and the TS glyphs written by them was 0.73.”
Please add the interpretation of this particular analysis.

There is an inverse correlation between the age group of participants with RS glyphs and a positive correlation with TS glyphs.

We have interpreted the correlation results in the discussion (lines 425-431).

Line 453-460
The reference is on point. So, it can be backed up with further explanation. If possible, please elaborate on how the present study explores the mentioned aspect (Line 460)

We have now elaborated how the study explored Dual Route Theory of Reading aloud (lines 471-479).

Line 131-132 (In-Text citation)
“Studies have indeed shown that the orthographic knowledge for a language form early on in a speaker’s life, and is a function of exposure through education and reading habits (Apel, 2011; Apel et al., 2006; Masterson & Apel, 2010).”
All other references citation in-text has been numbered with the exception of this.

In-text citation has been updated and numbered accordingly (line 133).

Line 611-612
Lond Rev Educ
The abbreviation of this journal in the journal homepage is mentioned as “The London Review of Education (LRE)”
Journal homepage: <https://uclpress.scienceopen.com/collection/2122ff6c-4fcf-4054-a1f0-3be2b1ac2eae#:~:text=The%20London%20Review%20of%20Education,sectors%20and%20phases%20of%20education>.
Journal article: <https://uclpress.scienceopen.com/hosted-document?doi=10.1080/13603110600574454>
Please revise accordingly.

Thank you for the feedback. Here the IEEE reference system is automatically formatting the journal article names in an abbreviated form. This necessarily need not be same as the abbreviation of the journal title from their web page.

Figure 2
In Figure 2, the schematic representation of the self-paced reading experiment shows the instruction page followed by a sentence in RS. If possible, please add a sentence in TS also.

We have added the TS sentence in Figure 2 now (Line 282).
--------------------------------------------------------------------------------------------------------------

**Replies to Reviewer 2 comments:**


Points to improve/Suggestions for further research:

In section 6 (lines 414-415), the paper states that the correlation between group type and script type confirms that sublexical knowledge is determined by the script one learns in elementary education. However, does this correlation imply causation or confirmation? This question is significant because the later discussion mentions that sublexical orthographic knowledge could be restructured through continued exposure in the form of writing practice. There seems to be some conflict in clarity there.

It seems that there is a causation between group type and sublexical knowledge, because the age of acquisition of the script seems to govern the script that one uses to write in the production task. However, the two outliers indicate that the script for writing (and hence the sublexical knowledge) is an updatable skill. So, it seems like the relationship that we have discovered is really between sublexical knowledge and exposure/practise of the script. This is further discussed in lines 441-458.

In addition to writing being a production skill, it also differs from reading in terms of modality. Writing also has a motor processing component to it in addition to retrieval of orthographic representations. The paper briefly mentions age being a possible factor in writing speed in older participants. Is it possible for this difference in modality to have other consequences when it comes to the choice or preference of script type apart from exposure/writing practice?

Thank you for the question. It is very relevant to think about the effect of the modality itself when choosing one script over the other. When reading, the script choice is rarely subjective as both the visual and print media overtly choose the script for the viewers/readers. As we see in our results, the reading speed is affected by age. Despite the senile slowdown, the RS was read faster than TS by the elderly population. When it comes to writing, we did not overtly ask the participants to pick any script. Still the elderly picked TS for writing and the young participants chose RS. The speed of writing was not calculated during the task, as we wanted to know which script the participant would select if not explicitly asked to choose between scripts. This is an indication of their sublexical orthographic knowledge (Apel, 2011)

. The effect of modality here could be then the overt and covert choices the participants get to make. The overt choices are mostly a reflection of their lexical orthographic knowledge seen while reading tasks and the covert choices are reflection of their sublexical orthographic knowledge seen in the writing task. Reading, thus, is not a subjective activity in terms of choice of scripts as compared to writing, where the reader consciously or sub consciously gets to choose the script.

It would have been helpful if lexical and sublexical orthographic knowledge were explained using examples/language data.

We have added examples from language data (lines 126-133).

Similarly, more explanation for certain memory and processing related terms such as retrieval would be helpful for a reader who may be new to the area.

We added the explanation in the text (lines 181-186).

Is it possible that changes made in the script reform were also keeping in view that these revisions would simplify the script in terms of the cognitive load on the user? The paper passingly suggests otherwise. However, do you think it would be right to assume that reforms with such pragmatic intentions would naturally result in a simpler yet more efficient orthographic system?

The available historic discourse indicates that the government’s decision was made from a policy perspective, specifically to achieve a standard script in Malayalam. We cannot say for certain that this reform has (or has not) resulted in a simpler or efficient orthographic system for the language users, especially the elderly, since it appears that the ease of use is largely practice and exposure-based phenomenon. It must be admitted that the perceptual load for the TS-educated user could decrease due to less phonemic density, but it must be discounted that the cognitive load would for such a user would go up. This is because for a learner who had their elementary education in TS, their schema and sublexical knowledge would be formed by the glyphs and ligatures in TS. An addition of RS glyphs and altered way of writing would increase the cognitive load due to the limited working memory capacity. The newly learnt RS script and the available schema in TS would result in crowding of the cognitive capacity leading to less efficient writing or reading activity by the language user.

I would also like to alert the authors of some minor proof-reading errors that need to be corrected.

Thank you for pointing this out. We have corrected the proof-reading errors in the manuscript.
